# Functional Evaluation of Endoscopic Treatment of Ischiofemoral Impingement: Case Reports

**DOI:** 10.1055/s-0042-1742343

**Published:** 2022-02-15

**Authors:** Bruno Silva Tavares, Ricardo Mendonça de Paula, Lucas Ricci Delevedove, Pedro Ivo Ferreira Favaro, Leonardo Santa Cruz Nogueira, Leandro Alves de Oliveira

**Affiliations:** 1 Ortopedia e Traumatologia, Hospital Estadual de Urgências da Região Noroeste de Goiânia Governador Otávio Lage de Siqueira (HUGOL). Secretaria de Estado da Saúde, Goiânia, GO, Brasil; 2 Grupo de Quadril do Hospital Estadual de Urgências da Região Noroeste de Goiânia Governador Otávio Lage de Siqueira (HUGOL), Secretaria de Estado da Saúde, Goiânia, GO, Brasil; 3 Hospital Regional de Araguaina, TO, Brasil; 4 Grupo de quadril do Departamento de Ortopedia e Traumatologia, Hospital Estadual de Urgências da Região Noroeste de Goiânia Governador Otávio Lage de Siqueira (HUGOL), Secretaria de Estado da Saúde, Goiânia, GO, Brasil

**Keywords:** femur, hip, ischium

## Abstract

Ischiofemoral impingement (IFI), although infrequent, should be thought of as one of the causes of deep gluteal pain syndrome. Difficulty in establishing a diagnosis and inaccurate clinical examination can be associated with the small number of case reports in the literature. The initial IFI treatment uses conservative measures, and surgical treatment is infrequent. The following is a case report of four adult patients, all female, diagnosed with IFI, with unsuccessful conservative treatments, in whom endoscopic resection of the smaller trochanter was performed with good results.

## Introduction

Ischiofemoral impingement (IFI), although infrequent, should be thought of as one of the causes of deep gluteal pain syndrome. The first description of IFI occurred in 1977 when, after total hip arthroplasty, three patients reported residual pain. 1 Recently, this type of impingement has been identified as a cause of pain even in patients without a history of trauma or hip surgeries. 2

The difficulty in establishing the clinical diagnosis of IFI stems from vague complaints from patients, usually related to deep gluteal pain, and inaccurate clinical examination, although the latter is necessary for impingement detection. 3 Surgical treatment is infrequent, with only 5% of patients requiring this type of intervention. 4

In cases refractory to conservative treatment, alternatives have been suggested. Therefore, this paper presents the case report of four adult patients diagnosed with IFI, in which there was no success with conservative treatment, and endoscopic resection of the smaller trochanter was performed.

## Case Report

The present study was submitted to the ethics committee with registration at Plataforma Brasil.

We included four female patients diagnosed with IFI: patient 1 was 35 years old and underwent endoscopic resection of the minor trochanter after 1 year of lumbar arthrodesis surgery; patient 2 was 53 years old and underwent endoscopic resection of the minor trochanter after 5 months lumbar arthrodesis postoperative; patient 3, 28 years old, and patient 4, 25 years old, had no previous surgeries.

The patients arrived at the orthopedic service complaining of chronic hip pain, and reported having been through several orthopedists without resolution of the condition. For functional evaluation of the hip, the *Harris Hip Score* 5 was applied before and after surgery. The preoperative score ranged from 49 to 68, with an average of 59 points. All patients scored less than 70 points in the preoperative period, which is considered a poor result, demonstrating limitations and exuberant pain, associated with a lower physical performance and quality of life. After 6 months postoperatively, the *Harris Hip Score* was applied again, ranging from 91 to 98 points, with an average improvement of 58.2%, which is considered an excellent result.

During the physical examination, the Long Steps test was performed, in which the patient is asked to make strides from 30 centimeters to 50 centimeters, in which the patient reported gluteal pain when performing such displacement. The provocative extension maneuver was also performed, and was associated with adduction and external rotation in lateral decubitus. 6 All patients presented with pain when performing the tests. After the endoscopic procedure, the pain was resolved.

**Fig. 1:**
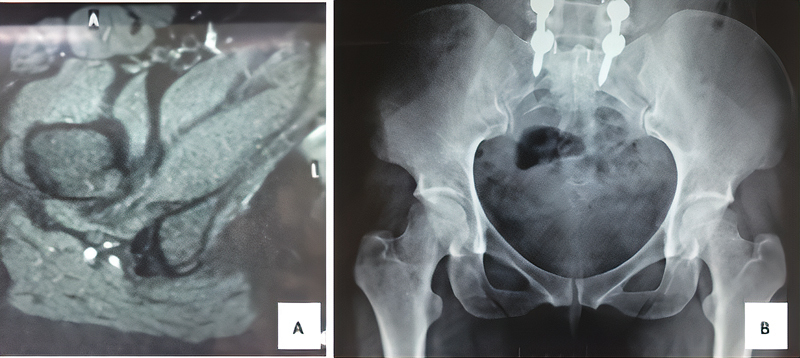
Preoperative nuclear magnetic resonance imaging ( **A** ) and radiography ( **B** ) of patient 1.

**Fig. 2:**
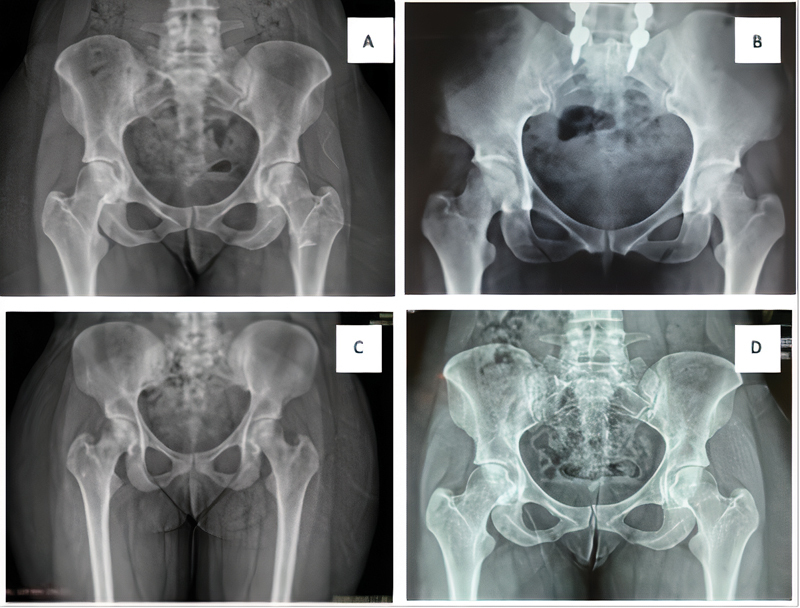
Preoperative radiographs of patients 1 ( **A** ), 2 ( **B** ), 3 ( **C** ) and 4 ( **D** ).

The imaging tests ( Figs. 1 and 2 ) included radiography and magnetic resonance imaging (MRI). Preoperative examinations showed no joint alterations or other deformities, only a decrease in the ischiotrochanteric space. Furthermore, there was no evidence of cysts in the ischium, which are characteristic of chronic lesions. The MRI examinations focused on characterizing the cause of deep gluteal pain with accuracy by scanning the quadratus femoris muscle, acetabular labrum, hip joint cartilage and other muscles with their respective tendons. This exam can also be done with an axial cut measuring the ischiofemoral interval, which has 18 to 24 mm as normal value. 7 The MRI scans did not show labral and/or chondral lesions or musculotendinous alterations in any of the patients. However, the presence of edema in the quadratus femoris muscle was evident, mainly visualized in the axial section, which characterizes IFI. After the diagnosis of IFI, all of the patients underwent conservative treatment with non-steroidal anti-inflammatory drugs, physiotherapy, and opioid analgesia for 6 months, without success.

**Fig. 3:**
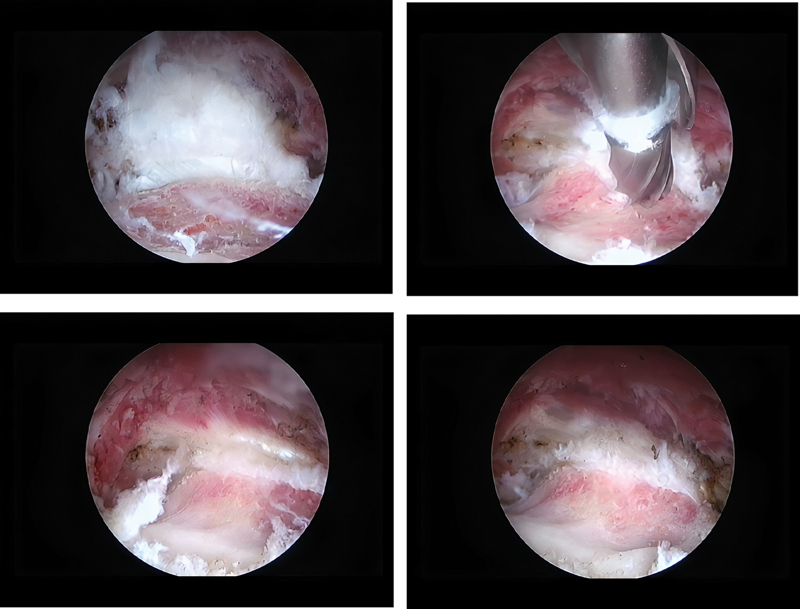
Endoscopic images of the lesion and resection of the patient 3's minor trochanter.

**Fig. 4:**
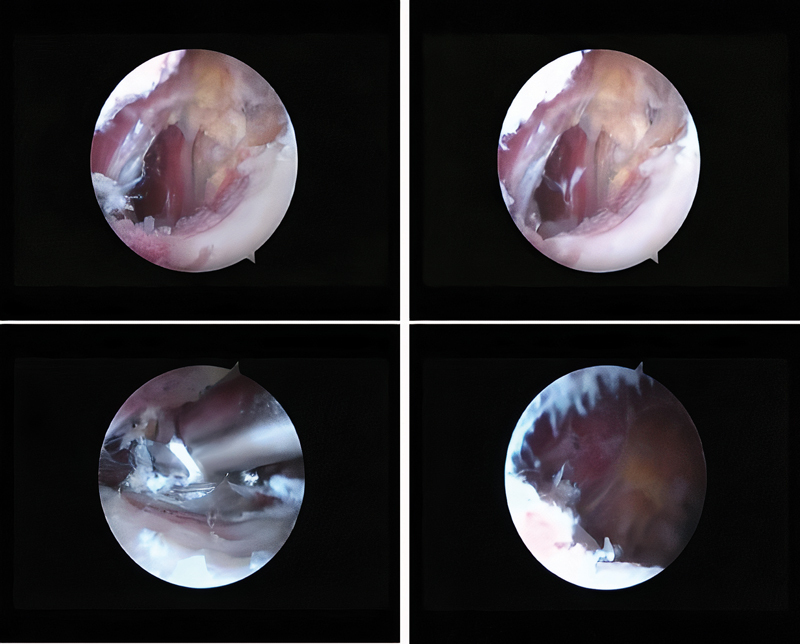
Endoscopic images of the lesion and resection of the patient 4's minor trochanter.

After this period, surgical treatment was performed. The technique described by Jo and O'Donnell 8 had the patient in supine position in a traction table with the affected limb positioned in maximum flexion, external rotation and adduction to anteriorize the small trochanter. Two previous surgical portals were made, the first one in the topography of the small trochanter, and the second one in a closer position using as reference the top of the large trochanter and a line perpendicular to the anterosuperior iliac spine. After endoscopic dissection of the small trochanter, a complete resection was performed, without reinserting the iliopsoas tendon ( Figs. 3 and 4 ).

**Fig. 5:**
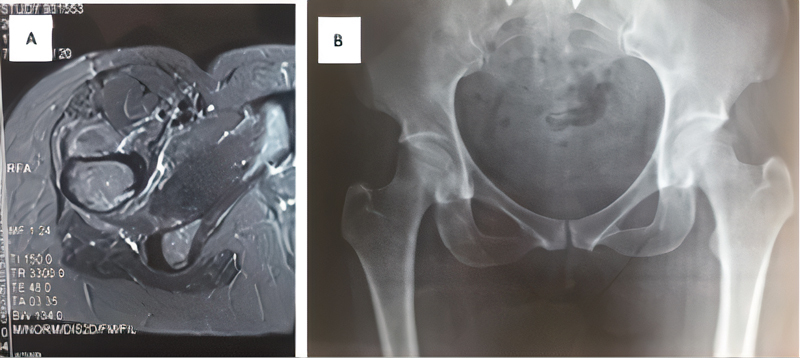
Nuclear magnetic resonance imaging ( **A** ) and radiography ( **B** ) with evident improvement of edema in the quadratus femoris muscle of patient 4.

After surgical treatment, an NMRI scan was repeated, in which the improvement of the edema in the quadratus femoris was observed ( Fig. 5 ). After 6 months of follow-up, all patients reported improvement of pain and functional capacity, returning to their usual activities without complaints.

## Discussion

Most IFI cases are in women, given their predisposition due to factors such as femoral anteversion and increased cervicodiaphyseal angle, wider pelvis, which are all typical of the female morphology. The diagnosis is commonly made by MRI scan, which is fundamental to identify changes in the ventral portion of the quadratus femoris muscle. 4 An acceptable size for the ischiofemoral space of 18 to 24 mm is considered, within which the disease is not characterized. 7

Pain relief was obtained after the minor trochanter resection in 3 patients with complaints of residual pain after total hip arthroplasty, as described by Johnson in 1977. 1 In 2008, Patti et al. 9 established the relationship between ischiofemoral narrowing as a potential source of hip pain in patients without previous history of trauma or surgery. By means of radiography and MRI, the authors observed a severe narrowing of the ischiofemoral space, an edema of the quadratus femoris muscle, and cystic alterations of the ischium.

Tosun et al. 10 evaluated 50 patients with hip pain and quadratus femoris muscle edema, and concluded that IFI was more common in women, aged between 51 and 53 years. Torriani et al. 3 found evidence that isolated changes in the quadratus femoris muscle could serve as a warning for a possible narrowing of the ischiofemoral space, and should be considered in the radiological evaluation. Ali et al. 11 reported the case of a 17-year-old patient who presented posttraumatic hip pain, with posterior ischiofemoral narrowing, and a quadratus femoris muscle edema in the NMRI evaluation. Surgical resection of the minor trochanter was performed, resulting in hip pain relief.

Yanagishita et al. 12 reported the case of a 31-year-old woman complaining of hip pain, with no history of trauma, evidence of ischiofemoral space narrowing, and an edema in the quadratus femoris muscle. After clinical evaluation, and radiological and NMRI examinations, the patient underwent conservative treatment, with noted functional improvement after 3 months of treatment, without undergoing surgical intervention.

Hatem et al. 6 evaluated the results of the endoscopic treatment, with partial resection of the lower trochanter, in five patients with IFI, and observed that the mean *Harris Hip Score* increased from 51.3 points preoperatively to 94.2 points in the postoperative period, an improvement of 83%.

In the cases reported here, an endoscopic resection of the minor trochanter was performed, with the ischiofemoral space being reestablished, thus ceasing the aggression to the quadratus femoris muscle. Endoscopic treatment is noted as one of the most important therapeutic options for the treatment of IFI. Furthermore, the fact that two of the patients in this case study had previous lumbar arthrodesis, without pain improvement even after being through several professionals, with inconclusive diagnoses, suggests that IFI should also be placed on the list of differential diagnoses of lumbosciatalgia.
