# Supplementary material for: Functional Evaluation of Endoscopic Treatment of Ischiofemoral Impingement: Case Reports
Source: Rev Bras Ortop (Sao Paulo). 2022 Feb 15;59(Suppl 1):e43–8. doi: 10.1055/s-0042-1742343 (PMC11254445; doi:10.1055/s-0042-1742343)
Supplement: Related texts

# Research article: Avaliação funcional do tratamento endoscópico do impacto isquiofemoral: Relatos de caso

**Keywords:** fêmur, ísquio, quadril

## Abstract

O impacto isquiofemoral (IIF), apesar de infrequente, deve ser pensado como uma das causas da síndrome da dor glútea profunda. A dificuldade em se estabelecer o diagnóstico de IIF e os exames clínicos imprecisos podem ser associados à pequena quantidade de relatos de caso na literatura. O tratamento inicial de IIF utiliza medidas conservadoras, sendo o tratamento cirúrgico pouco frequente. A seguir, está o relato de caso de quatro pacientes adultas com diagnóstico de IIF sem sucesso com tratamento conservador, nas quais realizou-se a ressecção endoscópica do trocanter menor com bons resultados.

## Introdução

O impacto isquiofemoral (IIF), apesar de infrequente, deve ser pensado como uma das causas da síndrome da dor glútea profunda. A primeira descrição do IIF ocorreu em 1977, quando três pacientes relataram dor residual após artroplastia total do quadril. 1 Recentemente, esse tipo de impacto foi identificado como uma causa de dor em pacientes sem histórico de trauma ou cirurgias do quadril. 2

A dificuldade em se estabelecer o diagnóstico clínico do IIF decorre das queixas vagas dos pacientes, normalmente relacionadas à dor glútea profunda, e exame clínico impreciso, embora esse último seja necessário para a detecção do IIF. 3 O tratamento cirúrgico é pouco frequente, e apenas 5% dos pacientes necessitam desse tipo de intervenção. 4

Em casos refratários ao tratamento conservador, alternativas têm sido sugeridas. Assim, este artigo apresenta o relato de caso de quatro pacientes adultas com diagnóstico de IIF, nas quais não se obteve sucesso com o tratamento conservador, e, portanto, foi realizada a ressecção endoscópica do trocanter menor.

## Relato de casos

O presente trabalho foi submetido ao comitê de ética com registro na Plataforma Brasil.

Foram incluídas quatro pacientes do sexo feminino diagnosticadas com IIF: paciente 1, com 35 anos, passou por ressecção endoscópica do trocanter menor 1 ano após a cirurgia de artrodese lombar; paciente 2, com 53 anos, passou por ressecção endoscópica do trocanter menor após 5 meses de pós-operatório de artrodese lombar; paciente 3, com 28 anos, e paciente 4, com 25 anos, ambas sem cirurgias anteriores.

As pacientes chegaram ao serviço de ortopedia com queixa de dor crônica no quadril, e relataram terem passado por diversos ortopedistas sem resolução do quadro. Para avalição funcional do quadril, foi aplicado o *Harris Hip Score* 5 antes e após a cirurgia. A pontuação obtida no pré-operatório variou de 49 a 68, com média de 59 pontos. Todas as pacientes totalizavam menos de 70 pontos no pré-operatório, o que caracteriza um resultado ruim, demonstrando limitações e quadro álgico exuberante, associados a um menor desempenho físico e qualidade de vida. Após 6 meses de pós-operatório, foi novamente aplicado o *Harris Hip Score* , que variou de 91 a 98 pontos, com uma melhora média de 58,2%, que foi considerado um resultado excelente.

No exame físico, foi realizado o teste dos passos largos, no qual é solicitado ao paciente realizar passadas de 30 centímetros a 50 centímetros de distância, no qual o paciente refere dor glútea ao realizar tal deslocamento. Realizou-se também a manobra provocativa de extensão, que é associada à adução e rotação externa em decúbito lateral. 6 Todos os pacientes cursaram com dor ao realizar os testes. Após o procedimento endoscópico, houve resolução das dores.

Os exames de imagem ( Figs. 1 e 2 ) incluíram radiografias e ressonância nuclear magnética (RNM). Os exames pré-operatórios não apresentaram alterações articulares e nem outras deformidades, apenas diminuição do espaço isquiotrocantérico. Além disso, não foi encontrada evidência de cistos no ísquio, que são característicos de lesões crônicas. Os exames de RNM foram focados na caracterização da causa da dor glútea profunda com exatidão, ao avaliar o músculo quadrado femoral, labrum acetabular, cartilagem da articulação coxofemoral e demais músculos com seus respectivos tendões. Pode-se também, através do corte axial, fazer a medição do IIF, cujo valor normal é de 18 a 24 mm. 7 A avaliação por RNM não evidenciou lesões labrais e/ou condrais e nem alterações musculotendíneas em nenhuma das pacientes. Porém, foi evidente a presença de edema no músculo quadrado femoral, principalmente visualizado no corte axial, o que caracteriza o IIF. Feito o diagnóstico, todas as pacientes passaram por tratamento conservador com anti-inflamatórios não esteroides, fisioterapia, e analgesia com opioide por 6 meses, sem sucesso.

**Fig. 1:** Ressonância nuclear magnética ( **A** ) e radiografia pré-operatórias ( **B** ) da paciente 1.

**Fig. 2:** Radiografias pré-operatórias das pacientes 1 ( **A** ), 2 ( **B** ), 3 ( **C** ) e 4 ( **D** ).

Após esse período, realizou-se o tratamento cirúrgico. Utilizou-se a técnica descrita por Jo e O'Donnell, 8 com paciente em decúbito dorsal em mesa de tração com o membro afetado posicionado em flexão, rotação externa e adução máximas com intuito de anteriorizar o pequeno trocanter. Foram feitos dois portais cirúrgicos anteriores, sendo o primeiro na topografia do pequeno trocanter e o segundo em uma posição mais proximal usando como referência o topo do grande trocanter e uma linha perpendicular à espinha ilíaca anterossuperior. Após feita a dissecção endoscópica do pequeno trocanter, realizou sua ressecção completa, sem reinserir o tendão do iliopsoas posteriormente ( Figs. 3 e 4 ).

**Fig. 3:** Imagens endoscópicas da lesão e da ressecção do trocanter menor da paciente 3.

**Fig. 4:** Imagens endoscópicas da lesão e da ressecção do trocanter menor da paciente 4.

Após o tratamento cirúrgico foi repetida a RNM, na qual foi observada a melhora do edema no quadrado femoral ( Fig. 5 ). Após 6 meses de acompanhamento, todas as pacientes relataram melhora da dor e da capacidade funcional, retornando as suas atividades habituais sem queixas.

**Fig. 5:** Ressonância nuclear magnética ( **A** ) e radiografia ( **B** ) com evidente melhora do edema no músculo quadrado femoral da paciente 4.

## Discussão

A maioria dos casos de IIF são em mulheres, dado sua predisposição decorrente de fatores como a anteversão femoral e ângulo cervicodiafisário aumentados, e pelve mais alargada, que são todos típicos da morfologia feminina. O diagnóstico é comumente feito por RNM, que é fundamental para identificar alterações no ventre muscular do quadrado femoral. 4 Considera-se um tamanho aceitável para o espaço isquiofemoral de 18 a 24 mm, dentro do qual não se caracteriza a doença. 7

O alívio da dor foi obtido após a ressecção do trocanter menor em 3 pacientes com queixas de dor residual após artroplastia total do quadril, como descrito por Johnson em 1977. 1 Em 2008, Patti et al. 9 estabeleceram a relação entre o estreitamento isquiofemoral como fonte potencial de dor no quadril em pacientes sem história prévia de trauma ou cirurgia. Por meio de radiografia e RNM os autores observaram um estreitamento severo do espaço isquiofemoral, um edema do músculo quadrado femoral e alterações císticas do ísquio.

Tosun et al. 10 avaliaram 50 pacientes com dor no quadril e edema do músculo quadrado femoral, e concluíram que o IIF foi mais comum em mulheres, com idade entre 51 e 53 anos. Torriani et al. 3 encontraram evidência de que alterações isoladas do músculo quadrado femoral poderiam servir de alerta para um possível estreitamento do espaço isquiofemoral, e deviam ser consideradas na avaliação radiológica. Ali et al. 11 relataram o caso de uma paciente com 17 anos que apresentou dor pós-traumática no quadril, com estreitamento isquiofemoral posterior, e edema do músculo quadrado femoral na avaliação por RNM. Foi realizada a ressecção cirúrgica do trocanter menor com resultante alívio da dor no quadril.

Yanagishita et al. 12 relataram o caso de uma mulher de 31 anos com queixa de dor no quadril, sem histórico de trauma, com evidência de estreitamento do espaço isquiofemoral e edema no músculo quadrado femoral. Após avaliação clínica, exames radiológicos e de RMN, a paciente foi submetida a tratamento conservador, com melhora funcional notável após 3 meses de tratamento, não sendo submetida a intervenção cirúrgica.

Hatem et al. 6 avaliaram os resultados do tratamento endoscópico, com ressecção parcial do trocanter menor, em cinco pacientes com IIF, e observaram que a média do *Harris Hip Score* aumentou de 51,3 pontos no pré-operatório para 94,2 pontos no pós-operatório, uma melhora de 83%.

Nos casos relatados aqui, foi realizada a ressecção endoscópica do trocanter menor, no qual se reestabeleceu o espaço isquiofemoral, cessando assim a agressão ao músculo quadrado femoral. O tratamento endoscópico é visto como uma das mais importantes opções terapêuticas para o tratamento do IIF. Além disso, o fato de duas das pacientes apresentarem artrodese lombar prévia, sem melhora da dor mesmo após passarem por vários profissionais com diagnósticos inconclusivos, sugere que o IIF também deve ser colocado na lista dos diagnósticos diferenciais de lombociatalgia.
